# Preparation of Esterified Bacterial Cellulose for Improved Mechanical Properties and the Microstructure of Isotactic Polypropylene/Bacterial Cellulose Composites

**DOI:** 10.3390/polym8040129

**Published:** 2016-04-12

**Authors:** Bo Wang, Dan Yang, Hai-rong Zhang, Chao Huang, Lian Xiong, Jun Luo, Xin-de Chen

**Affiliations:** 1 Key Laboratory of Renewable Energy, Guangdong Key Laboratory of New and Renewable Energy Research and Development, Guangzhou Institute of Energy Conversion, Chinese Academy of Sciences, Guangzhou 510640, China; 13546474299@163.com (B.W.); yangdan@ms.giec.ac.cn (D.Y.); zhanghr@ms.giec.ac.cn (H.Z.); huangchao@ms.giec.ac.cn (C.H.); xionglian@ms.giec.ac.cn (L.X.); 2 University of Chinese Academy of Sciences, Beijing 100049, China; 3 Research and Development Center of Xuyi Attapulgite Applied Technology, Chinese Academy of Sciences, Xuyi 211700, China; 4 Guangzhou Fibre Product Testing and Research Institute, Guangzhou 510220, China

**Keywords:** bacterial cellulose, isotactic polypropylene, composites, mechanical properties, esterification

## Abstract

Bacterial cellulose (BC) has great potential to be used as a new filler to reinforce isotactic polypropylene (iPP) due to its high crystallinity, biodegradability, and efficient mechanical properties. In this study, esterification was used to modify BC, which improved the surface compatibility of the iPP and BC. The results indicated that the cellulose octoate (CO) changed the surface properties from hydrophilic to lipophilic. Compared to the pure iPP, the tensile strength, charpy notched impact strength, and tensile modulus of the iPP/BC composites increased by 9.9%, 7.77%, and 15.64%, respectively. However, the addition of CO reinforced the iPP/CO composites. The tensile strength, charpy notched impact strength, and tensile modulus of the iPP/CO composites increased by 14.23%, 14.08%, and 17.82% compared to the pure iPP. However, the elongation at break of both the composites is decreased. The SEM photographs and particle size distribution of the composites showed improvements when the change of polarity of the BC surface, interface compatibility, and dispersion of iPP improved.

## 1. Introduction

Isotactic polypropylene (iPP) is an important commercial plastic that is widely used as a matrix component in packaging, construction, and other industrial applications due to the wide range of useful properties such as low density, high strength, chemical resistance, wear-resisting, and low price. However, the poor impact strength of iPP limits its applications [1,2,3,4,5]. There are a lot of research available using nano particles including calcium carbonate [6], montmorillonite [7], and cellulose [8] to reinforce the iPP.

Bacterial cellulose (BC) is a new biodegradable polymer that has a similar structure to plant cellulose and has great potential for materials, food, and medicine applications [9,10]. BC is made of a superfine fiber network, and has the advantages of artificially controlled synthesis, biodegradability, high purity, high crystallinity, high degree of polymerization, and good mechanical properties [11].

**Figure 1:**
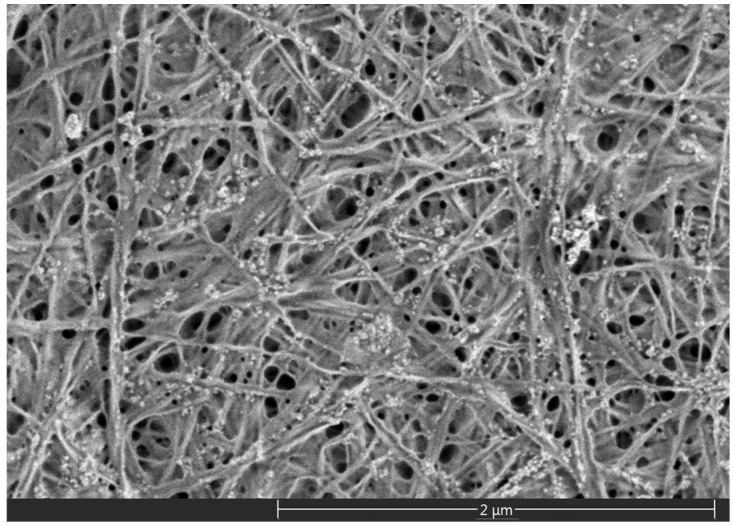
The micro topography of bacterial cellulose.

The initial physical form of BC is more fibrous than microcrystalline (Figure 1). Thus, the BC has a great potential to reinforce iPP as a starting material, since the fibrous nature of the BC can serve as a stress concentration when blended with iPP, which make the materials absorb a large amount of energy when facing external force, thus enhancing the mechanical properties of the composite [12]. These characteristics allow for the application of BC as a kind of new iPP filler, which has rarely been reported.

Generally, the polymer composites with excellent mechanical properties must have a multi-phase structure of microscopic or sub-microscopic morphology. The stability of the multi-phase system depends on the formation of the transition layer, the bonding of the interface, and the high viscosity of the macromolecular chain [13]. In particular, the bonding strength between the two polymers has a decisive influence on the mechanical properties of the composites.

However, the compatibility of hydrophilic BC and lipophilic iPP is poor because the molecular chain of BC has many hydrophilic hydroxyl groups, making the BC easier to gather in iPP, which can result in weak dispersion [14]. Moreover, when the composite is subjected to external forces, the stress cannot be effectively transmitted at the interface because of the poor interfacial compatibility between BC and iPP [15]. All of these factors hinder the mechanical properties of the composite. Currently, coupling agent modification, esterification, and biological modification are the most used methods to solve the interface issues of BC and iPP [16,17,18,19]. Among them, esterification has the advantages of being simple and convenient and having mild reaction conditions and a high degree of hydroxyl substitution on BC. The surface properties of BC change from hydrophilic to lipophilic after esterification [19].

In this study, through the esterified modification of BC, cellulose octoate (CO) was prepared. High-strength iPP/BC and iPP/CO composites were made, and the mechanical and micro-structural properties of the composites were characterized.

## 2. Materials and Methods

### 2.1. Materials

The BC film (Hainan Yida Food Industry Co., Ltd., Hainan, China) was flushed with large amounts of deionized water to neutralize the film, dried using a freeze-drying machine, and milled into powder (20~200 nm). The iPP S1003 with *M*_w_ = 4.1 × 10^5^ and *M*_w_/*M*_n_~4 was obtained from Beijing Yanshan Petrochemical Co. Ltd., Beijing, China. Pyridine and caprylyl chloride were purchased from Aladdin Industrial Corporation, Shanghai, China.

### 2.2. Preparation of CO

First, the BC powder and pyridine (*w/w*, 1:20) were dispersed in a flask with a stir bar at room temperature for 30 min, and the temperature of the system was then increased to 80 °C. When the temperature of the system stabilized, the caprylyl chloride (with the mass ratio of BC 30:1) was added dropwise with a dropping funnel. After 7 h, the solid product of the system was washed with ethyl alcohol and deionized water to neutralize the product and dried in air-oven for 12 h, and CO was obtained.

### 2.3. Characterization of CO

For the infiltration experiment, 0.1 g BC and 0.1 g CO were added separately in to the sample bottles containing 10 mL of water and 10 mL of toluene. After oscillation, both samples were kept at room temperature for 12 h.

Fourier transform infrared (FT-IR) was performed on a FT-IR spectrometer (Bruker Corporation, Tensor 27, Karlsruhe, Germany) taking 32 scans for each sample. The samples were dispersed in KBr. The sample with KBr was carefully dried before disk preparation and were subjected to FT-IR and immediately analyzed.

X-ray diffraction spectra were recorded from 2θ of 0° to 40° with a XRD diffraction instrument (PANalytical Company, Rigaku D/max-250, Eindhoven, Netherlands), with a Cu Kα radiation source at λ of 0.154 nm (10 kV, 100 mA).

### 2.4. Preparation of the iPP/BC and the iPP/CO Composites

The mixing weight ratio of the composites is shown in Table 1. Each component was mixed with a high-speed mixer (Laizhou Shenglong Chemical Machinery CO., Ltd. SHR-5A, Laizhou, China) for 5 min at the speed of 2000 rpm. The pellets of the composites were prepared using the twin-screw extruder (Nanjing Jieya Extrusion Equipment CO., Ltd. SHJ-20, Nanjing, China). The pellets were dried at 80 °C for 12 h, and the temperatures of each division increased to 170, 185, 195, and 200 °C. Finally, the standard test specimens of the composites were finally injection molded by an injection molding machine (Fomtec Machinery Co., Ltd., FT-150, Suzhou, China) the injection molding temperature and pressure were set to 170 °C and 15.5 MPa, respectively.

### 2.5. Mechanical Test

The tensile (testing speed, 20 mm/min) properties of the composites were tested with a universal testing machine (Z020, Zwick Roell Group, Ulm-Einsingen, Germany) at room temperature. The charpy notched impact strength of the composites was notched to a depth of 2 mm on the narrow side of the specimens using a notching machine (Ceast AN50, Instron, MA, USA) and measured with an impact tester (Ceast 9050, Instron, MA, USA) with 1J capacity at the maximum pendulum angle (150°) at room temperature. Ten replicates of each sample were tested with a mechanical test.

### 2.6. Scanning Electron Microscopy

Samples were viewed via SEM (Quanta 250 FEG, FEI, Hillsboro, OR, USA) at an accelerated voltage of 10 kV in order to observe the compatibility at the tensile fractured surfaces of the composites. An image analysis program (Nano measurer 1.2, Laboratory of surface chemistry and catalysis, Fudan University, Shanghai, China) was used to measure the particle size distribution of the samples from the micrographs. For each sample, more than 200 particles were examined [20].

## 3. Results and Discussion

### 3.1. Characterization of CO

#### 3.1.1. Infiltration of BC and CO

**Figure 2:**
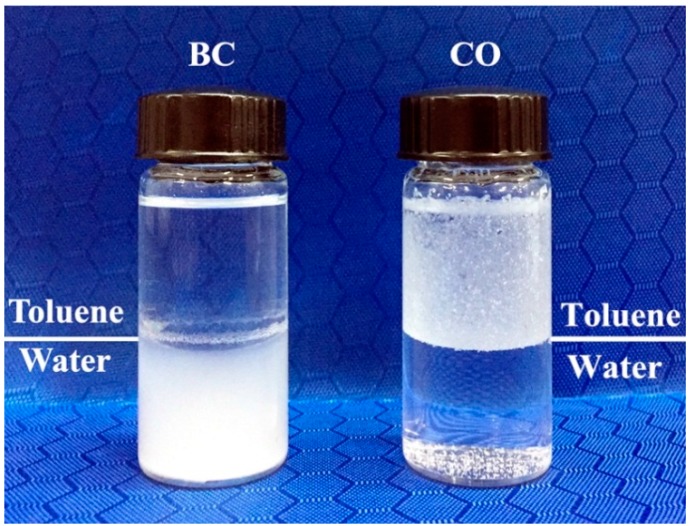
Infiltration experiment of BC and cellulose octoate (CO).

The infiltration experiment of BC and CO is shown in Figure 2. It can be seen in the left bottle that the BC can only scattered in the water, and in the right bottle most of the CO dispersed in the toluene. As we know, the polarity is higher for water than for toluene. Thus, BC can infiltrate the water. After esterification, the polarity of CO was greatly reduced, which allowed toluene to infiltrate the CO powder. This phenomenon can prove that esterification successfully changed the surface properties of BC from hydrophilic to hydrophobic.

#### 3.1.2. FT-IR Spectra of BC and CO

**Figure 3:**
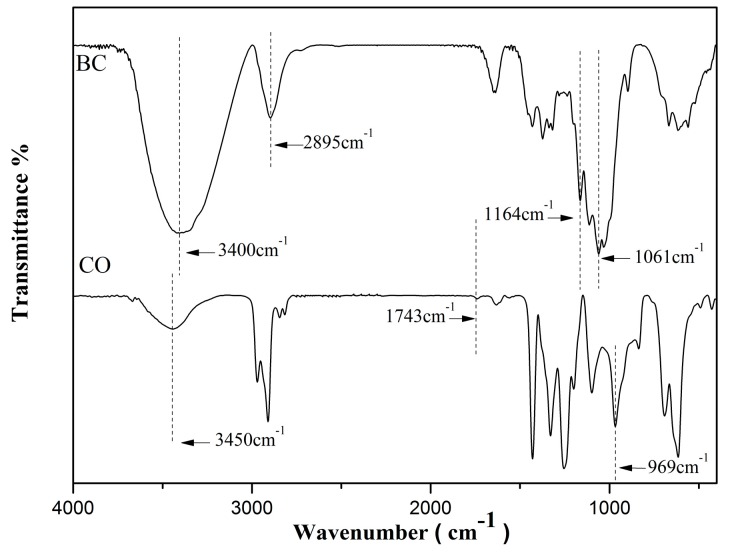
Fourier transform infrared (FT-IR) spectra of BC and CO.

The FT-IR analysis of BC and CO is shown in Figure 3. The O–H stretching vibration absorption peak at 3400 cm^−1^, the C–H bond stretching vibration absorption peak at 2895 cm^−1^, the C–O stretching vibration absorption peak at 1061 cm^−1^, and the asymmetric vibration absorption peak at 1164 cm^−1^ appeared in the spectra of BC [21].

Compared with that of BC, the spectrum of CO had several differences. After esterification, a new acyl group was added to the BC, as indicated in Figure 2. The CO spectrum showed the expected C=O group vibrations at 1743 and –CH_3_ at 969 cm^−1^. Those absorption peaks proved that the esterification reaction of the BC was successful [22]. The O–H stretching vibration absorption peak of the CO at 3600–3200 cm^−1^ moved to a high wavenumber, the peak intensity became weak, and the peak width became narrow, which indicated that the hydrogen bond structure of the BC molecules were weakened, and some hydroxyl groups were substituted after the esterified modification [23]. Additionally, the CO spectrum did not show absorption peaks at 1840~1750 and 1700 cm^−1^. This suggested that there was no reaction and that there was non-clean octoic acid in the product.

#### 3.1.3. X-ray Analysis of BC and CO

**Figure 4:**
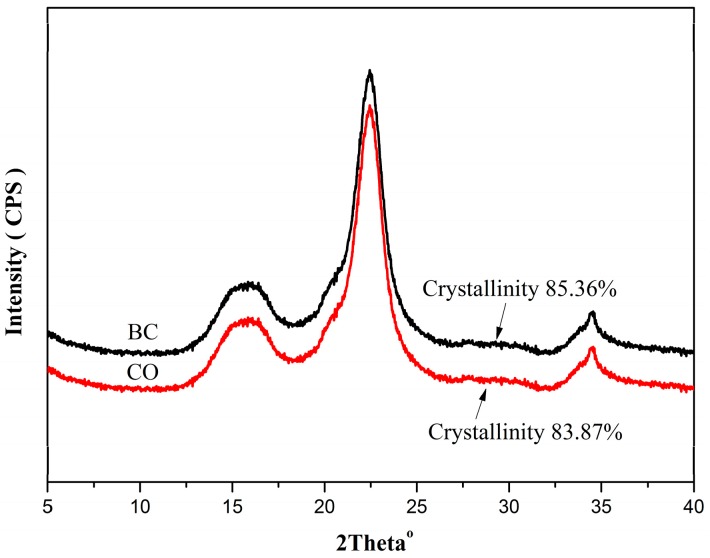
X-ray spectra of BC and CO.

The XRD spectra of BC and CO are illustrated in Figure 4. The XRD spectrum of CO was similar to that of BC, but the crystallinity of CO was lower. The main reason for this phenomenon may be that the surface modification of BC was carried out on the surface of the BC and not deep within the internal crystalline zone. The amorphous region of the BC surface was damaged, but the internal crystalline region was not affected, so the crystal shape of cellulose and the internal crystal structure were not damaged [24]. In the esterification reaction of BC, with the incessant forming and dissolving of CO, the reaction occurred in the amorphous region of the BC surface, the hydrogen bond of CO was weakened, the steric hindrance increased, and the crystalline structure of the BC was subject to certain destruction. Therefore, the original structure of the crystalline region was broken, and the crystallinity decreased [19].

### 3.2. Mechanical Properties of the Composites

**Figure 5:**
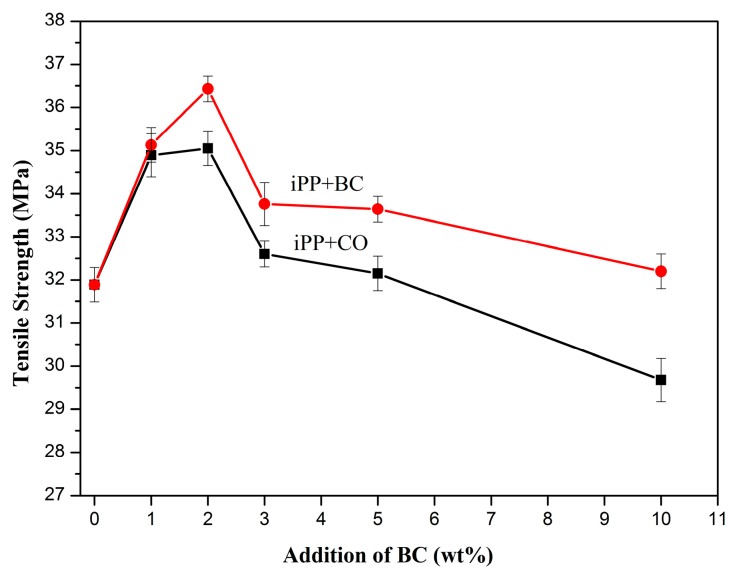
Tensile strength of the composites.

**Figure 6:**
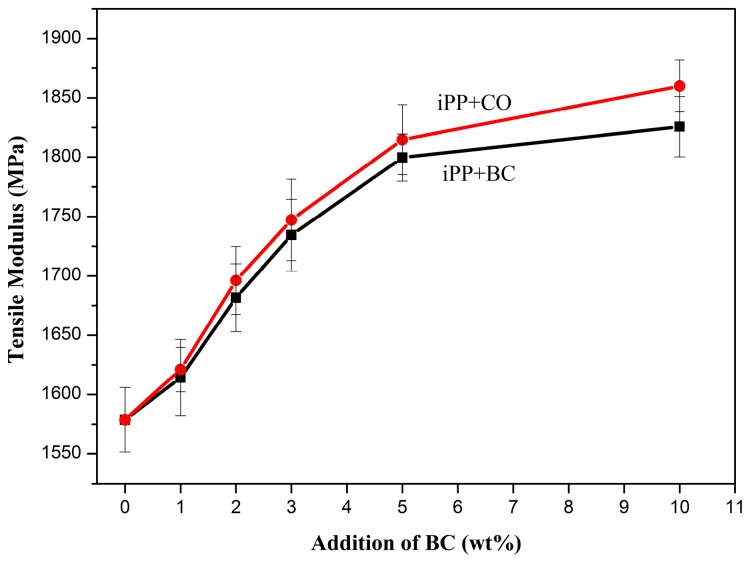
Tensile modulus of the composites.

**Figure 7:**
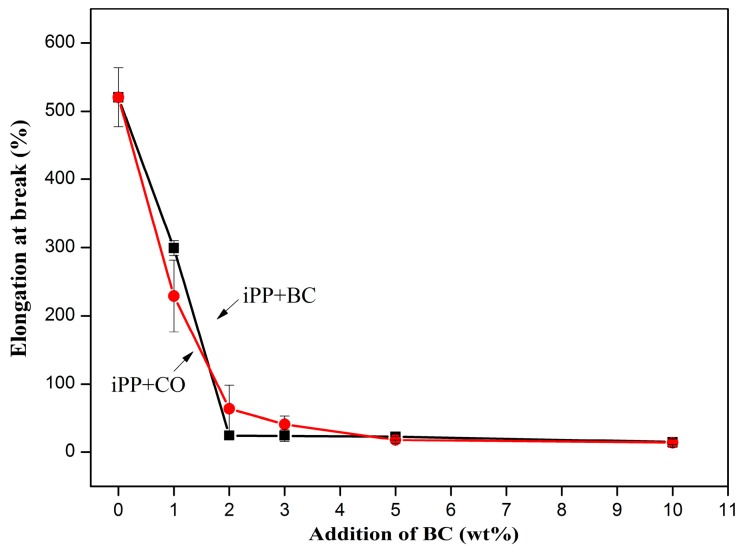
Elongation at break of the composites.

**Figure 8:**
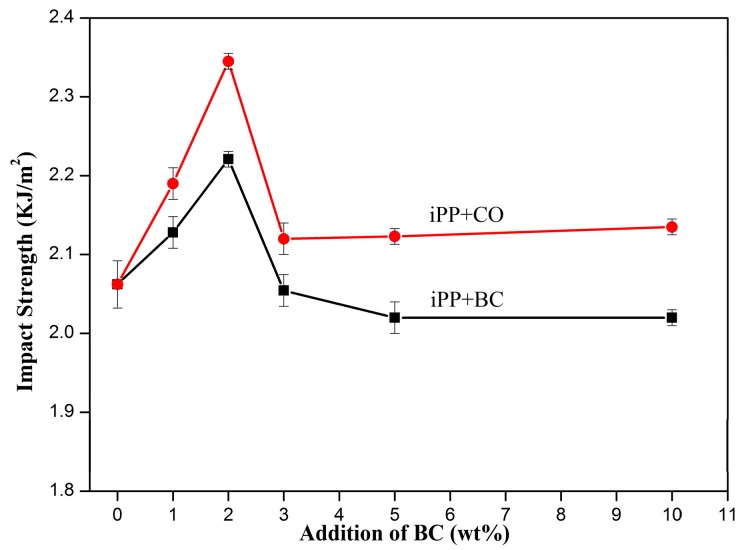
Charpy notched impact strength of the composites.

The mechanical properties at each experimental condition were determined, the mean values of the properties are shown in Figure 5, Figure 6, Figure 7 and Figure 8. The BC or CO provided an excellent measure of reinforcement to the composite.

It can be seen from Figure 5 that, when BC or CO was added from 0 to 2 wt %, the tensile strength of the composites increased. When the concentration was more than 2 wt %, the value was decreased. When BC or CO (2 wt %) was added, the tensile strength of the composites was at a maximum and increased from 31.89 to 35.05 and 36.43 MPa, respectively, representing a 9.9% and 14.23% increase over pure iPP. It was suggested that BC influenced the mechanical properties of the composites. The esterification improved the adhesions and interactions between the BC and iPP.

At the same time, with the addition of BC or CO, the tensile modulus of the composites was greatly increased from 1578.71 MPa to 1825.69 and 1859.99 MPa, respectively, representing a 15.64% and 17.82% increase over pure iPP (Figure 6), and the composites with CO were more superior to the composites with BC. The modification can change the molecular morphology of the polymer chains near the filler–polymer interphase [24].

The elongation at break (Figure 7) significantly declined with increased BC or CO concentrations. However, there was not much difference for composites with BC or CO. With increased filler concentrations, the steep decline of the elongation at break may prove that BC has a low elongation at break and may restrict the flow of polymer molecules past one another [25]. This behavior is typical of reinforced thermoplastics in general and has been reported by many researchers [25,26,27].

Similar to the trend of the tensile strength of composites, when BC or CO (2 wt %) was added, the charpy notched impact strength (Figure 8) of the composites was maximized and increased from 2.06 to 2.22 and 2.35 KJ/m^2^, respectively, representing a 7.77% and 14.08% increase over pure iPP. The notched impact behavior was controlled to a greater extent by factors affecting the propagation of fracture that was initiated due to a stress concentration at the notch tip.

According to the theory of the silver grain shear zone [28], BC or CO particles can cause the iPP to produce silver grains and shear band, which absorb a high amount of energy [29]. At the same time, the particles can stop the crack growth, purification, and termination of the silver grain. Because the particles had a smaller particle size, when the particle content in the iPP increased, the number of particles increased, the distance decreased, and the mutual interference of the silver grain particles was more obvious. It was easier to induce the iPP shear yielding and plastic flow, which can absorb energy [30]. Thus, the impact strength of the composites increased. However, when particle concentrations increased, BC or CO particle agglomeration occurred, producing defects and forming stress concentration points in the composites. The composites ‘declined ability to withstand external damage can decrease the performance of the impact strength.

The mechanical properties of the composites depended on the strength of the polymer, the filler, and the bond strength of the interface between the polymer and filler. The mechanical properties of the iPP/CO composites with the same ratio were generally better than those of the iPP/BC composites. It was proven that with the formation of CO, the iPP interfacial bonding strength was higher. The esterification modifications effectively enhanced the interface compatibility of iPP and BC.

### 3.3. Scanning Electron Microscopy Observations

**Figure 9:**
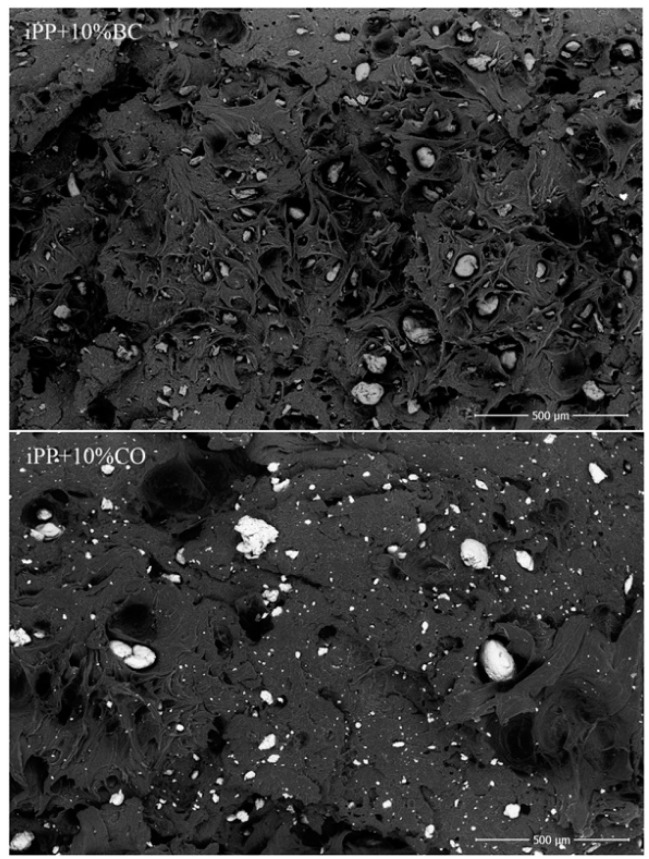
SEM photographs of the composites tensile fractured surface.

The SEM photographs of the composites tensile fractured surface are shown in Figure 9. It can be seen that the tensile fractured surface of the composites was divided into two phrases, and both the BC and CO agglomerated in the iPP matrix. Conversely, when BC was added to the iPP, holes remained in the smooth and clean matrix surfaces, and dispersion of the BC was poor, indicating poor compatibility between the iPP and BC. However, when CO was added, the remaining holes were smaller, which illustrated improved compatibility. The good compatibility between the iPP and CO resulted in better stress transfer and superior mechanical properties. As expected, by changing the polarity of the BC surface, the free energy of the BC surface decreased, the interface compatibility improved, and the iPP could effectively adhere to the BC surface. The good compatibility between the iPP and CO contributed to forming a strong interfacial layer that altered the local stress distribution [31]. This explained why the mechanical properties of the iPP/CO composite were better than the iPP/BC composite.

### 3.4. Particle Size Distribution of Bacterial Cellulose (BC) or Cellulose Octoate (CO) in the iPP Matrix

**Figure 10:**
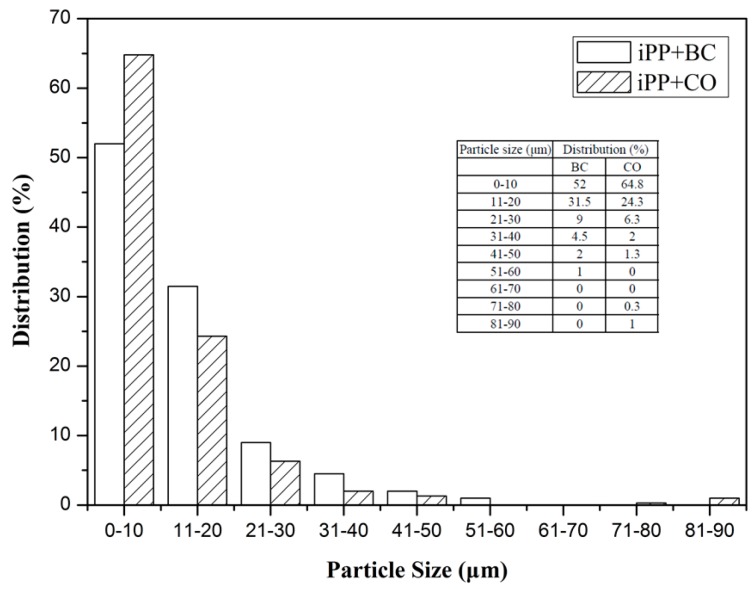
Particle size distribution of BC or CO in the isotactic polypropylene (iPP) matrix.

The particle size distribution of BC and CO in the iPP matrix was intensively studied. It can be seen from Figure 10 that the particle size of CO in the iPP matrix was significantly smaller than BC, and the dispersion was greatly improved. Recent studies showed that the larger particle sizes allow defects to easily form in the iPP matrix, and, as the particle sizes decrease, the specific surface of the particles increases, and the contact interface between the particles and matrix increases. Therefore, when the composite was subjected to external forces, it produced more micro cracks and plastic deformation, which can absorb more impact energy, which indicates good mechanical properties [32,33,34,35,36]. Consistent with the SEM result, this phenomenon provided evidence to explain why the mechanical properties of the iPP/CO composite were better than those of the iPP/BC composite.

## 4. Conclusions

CO was successfully prepared using caprylyl chloride as an esterifying agent, which decreased the hydrophilicity and crystallinity. The general trend of the mechanical properties of the composites demonstrated that the iPP/CO composite was better than the iPP/BC composite. Specifically, when BC or CO (2 wt %) was added, the tensile strength was maximized and increased by 9.9% and 14.23%, and the charpy notched impact strength was maximized and increased by 7.77% and 14.08% over the pure iPP. With the addition of BC or CO, the tensile modulus of the composites was greatly increased by 15.64% and 17.82%, and the elongation at break significantly declined with increased BC or CO concentrations, but there was little difference between the composites with BC or CO. The SEM photographs and particle size distribution of the composites showed improvements for BC when the polarity of the BC surface, the interface compatibility, and dispersion of iPP changed. This phenomenon provided evidence to explain why the mechanical properties of the iPP/CO composite were better than the iPP/BC composite.

## Figures and Tables

**Table 1:** Formula of the composites.

Sample	Isotactic Polypropylene (iPP) (wt %)	Bacterial Cellulose (BC) (wt %)	Cellulose Octoate (CO) (wt %)
iPP	100	–	–
iPP + 1% BC	100	1	–
iPP + 2% BC	100	2	–
iPP + 3% BC	100	3	–
iPP + 5% BC	100	5	–
iPP + 10% BC	100	10	–
iPP + 1% CO	100	–	1
iPP + 2% CO	100	–	2
iPP + 3% CO	100	–	3
iPP + 5% CO	100	–	5
iPP + 10% CO	100	–	10
